# A Cue from the Unconscious – Masked Symbols Prompt Spatial Anticipation

**DOI:** 10.3389/fpsyg.2012.00397

**Published:** 2012-10-12

**Authors:** Heiko Reuss, Andrea Kiesel, Wilfried Kunde, Peter Wühr

**Affiliations:** ^1^Department of Psychology III, Julius-Maximilians-Universität WürzburgWürzburg, Germany; ^2^Department of Psychology, Technische Universität DortmundDortmund, Germany

**Keywords:** endogenous shifts of attention, anticipation, unconscious processing, spatial cueing, masked priming

## Abstract

Anticipating where an event will occur enables us to instantaneously respond to events that occur at the expected location. Here we investigated if such spatial anticipations can be triggered by symbolic information that participants cannot consciously see. In two experiments involving a Posner cueing task and a visual search task, a central cue informed participants about the likely location of the next target stimulus. In half of the trials, this cue was rendered invisible by pattern masking. In both experiments, visible cues led to cueing effects, that is, faster responses after valid compared to invalid cues. Importantly, even masked cues caused cueing effects, though to a lesser extent. Additionally, we analyzed effects on attention that persist from one trial to the subsequent trial. We found that spatial anticipations are able to interfere with newly formed spatial anticipations and influence orienting of attention in the subsequent trial. When the preceding cue was visible, the corresponding spatial anticipation persisted to an extent that prevented a noticeable effect of masked cues. The effects of visible cues were likewise modulated by previous spatial anticipations, but were strong enough to also exert an impact on attention themselves. Altogether, the results suggest that spatial anticipations can be formed on the basis of unconscious stimuli, but that interfering influences like still active spatial anticipations can suppress this effect.

## Introduction

Humans can give priority to spatial locations where behaviorally relevant stimuli occur, a process referred to as spatial attention. Such orienting of attention can happen in two different ways, either *exogenously* driven or *endogenously* controlled (e.g., Posner, 1980; Jonides, 1981; Posner and Cohen, 1984; Müller and Rabbitt, 1989; Yantis and Johnson, 1990; Yantis and Jonides, 1990; Theeuwes, 1991; Folk et al., 1992). On the one hand, exogenous orienting of attention is induced by particular events in the environment. Here, anticipation plays a role insofar as only events that are behaviorally relevant are able to capture attention. If, for example, participants search for targets that abruptly onset on a screen, cues that abruptly onset automatically capture attention (Folk et al., 1992). Interestingly, if participants anticipate particular behaviorally relevant features, cues that owe these features grab attention automatically, even if they are overall not predictive for the target location.

On the other hand, humans can deliberately orient attention to certain locations in space, or in Helmholtz’s words, “it is possible, simply by a conscious and voluntary effort, to focus the attention on some definite spot in an absolutely dark and featureless field” (von Helmholtz, 1866, cited after Yantis, 1998, p. 225). Typically, such conscious efforts are suggested to the participant by some symbolic cue presented in the center of vision (Posner, 1980; Posner et al., 1980). Again, anticipation is a necessary process for such cues to work, but at a different point in time, namely after rather than before cue presentation. Only if subjects anticipate targets at the cued location will central cues leave a trace in performance. Endogenously controlled shifts of attention are only executed when the target in fact appears at the cued location more often than not and is thus anticipated there. Perhaps anticipation of the target location is what we typically describe as cueing effects or validity effects: faster response times (RT) to targets at validly cued compared to invalidly cued locations.

The distinction between these two forms of orienting of attention and also their dependency on awareness is nicely illustrated in a study by McCormick (1997). The cues in this experiment were peripheral bars that were either visible or masked. Critically, the target appeared at the opposite location of the cue in 85% of the trials, so that participants would anticipate the target at the non-cued location. McCormick reasoned that when a cue appeared, it would at first capture attention exogenously. However, this exogenous cue could then be used strategically by the participants to endogenously shift their attention to the opposite location, where they anticipated the target. McCormick found that with visible cues, participants were indeed reorienting their exogenously captured attention in anticipation of the target. When the cues were masked, however, performance was better when the target appeared at the location of the cue. This indicates that the masked cues were able to exogenously capture attention, but that the participants were not able to voluntarily reorient their attention when the cue was masked. Subsequent work has confirmed many times that masked cues trigger exogenous shifts of attention (McCormick, 1997; Lambert et al., 1999; Scharlau, 2002; Ivanoff and Klein, 2003; Scharlau and Ansorge, 2003; Scharlau and Neumann, 2003; Ansorge and Neumann, 2005; Ansorge and Heumann, 2006; Mulckhuyse et al., 2007; for a review, see Mulckhuyse and Theeuwes, 2010).

While the possibility of exogenous cues to work outside of awareness is in line with classical theories of automaticity and control (Atkinson and Shiffrin, 1968; Posner and Snyder, 1975), the more intriguing question is the relation of endogenously controlled orienting of attention and consciousness. In recent years, a steadily growing field of research is concerned with this relation of consciousness and cognitive control processes. For example, it was shown that the activation of task sets, a typical instance of cognitive control, can be triggered unconsciously by masked task cues (Mattler, 2006; Lau and Passingham, 2007; Reuss et al., 2011a). Also, there are findings that inhibition, a cognitive control process that is oftentimes conceptualized as the functional opposite of attention, can be induced unconsciously. When participants were presented with masked nogo-signals or masked stop signals, they tended to respond slower than without such a signal or they even inhibited their response altogether (van Gaal et al., 2008, 2009; Hughes et al., 2009). These findings suggest that the link between consciousness and cognitive control may not be as obligatory as traditional views of consciousness and control propose. As the focusing of attention on relevant information is regarded as one of the most elementary executive functions (Smith and Jonides, 1999), insights into the role of cue awareness in this process are essential for an understanding of the functional role of consciousness and different aspects of cognitive control.

Interestingly, however, findings regarding the role of awareness and endogenously controlled shifts of attention are scarce. As noted, McCormick (1997) found that cue awareness is necessary to perform shifts of attention in direction opposite to that indicated by a peripheral cue. Note, however, that subjects in that study had to first countermand the impact of a peripheral cue before subsequently attending to a new location. It remains therefore an open question whether masked central cues would have the power to induce shifts of attention when such countermanding is not needed. In fact Reuss et al. (2011b) found preliminary evidence for the orienting of attention by masked central cues provided attention has not already been grabbed by another event. However, this finding is preliminary due to the specific type of cues used, namely arrows (for a similar study with eye gaze cues, see Al-Janabi and Finkbeiner, 2012). Arrow cues and other stimuli such as eye gaze and hand gestures carry an over learned spatial meaning. Most crucially, they were found to successfully capture attention even when they were not informative regarding the target location, which is in fact a hallmark of a reflexive rather than voluntary orienting of attention (Eimer, 1997; Hommel et al., 2001; Tipples, 2002; Friesen et al., 2004; Gibson and Bryant, 2005; Stevens et al., 2008; Pratt et al., 2010).

Given these limitations of previous research, the present study explored if symbolic cues that carry no inherent spatial meaning have the power to bias attention without cue awareness. To study this, we presented letters that indicated the locations of the target stimuli. These cue letters were presented masked or unmasked with the presence or absence of masks changing randomly from trial to trial. The primary question was if central cues were able to impact on attention at all when they are presented unconsciously.

The experimental protocol allowed us to study another debated question regarding the effects of masked stimuli, namely carry-over effects from one trial to the next trial. A well-known sequential effect is the so-called Gratton-effect, which deals with the influence of the congruency of the previous trial on the congruency effect in the current trial. Typically, congruency effects are smaller after trials with incongruent primes than after trials with congruent primes. Several studies found such carry-over effects when primes in the preceding trial were visible but not when they were invisible (Greenwald et al., 1996; Kunde, 2003; Frings and Wentura, 2008; Ansorge et al., 2011), though under certain circumstances even invisible primes might prompt such carry-over effects (van Gaal et al., 2010). Here, we will investigate if a cue is able to impact on the next trial depending on its visibility and the visibility of the next cue. To this end, we will analyze if the size of the validity effects is modulated by these two factors. There are reasons to expect this, though the type of impact is admittedly less clearly predictable. On the one hand, one may argue that strategies from the processing of visible cues are simply transferred to masked trials (cf. Klapp and Haas, 2005). Consequently, the impact of masked cues should increase the more recent (ideally in the last trial) a visible cue had been encountered. On the other hand, one may assume that attentional orienting by visible cues is much stronger than that by masked cues (which is in fact what we found). Perhaps masked cues have a better chance to impact on performance the less attention is still influenced by a preceding visible cue. To specifically investigate if spatial anticipations are still active in the next trial, we will compare validity effects when the cued location repeats in contrast to when the cued location switches. Persisting spatial anticipations would result in larger validity effects when the cued location repeats compared to when the cued location switches.

## Materials and Methods

### Experiment 1

This experiment is based on the spatial cueing paradigm by Posner (1980), with centrally presented cues that indicate a lateral location, and a target display that either includes a target or consists solely of distractors. To make sure that any shifts of attention are truly endogenous, we used letters as cues which are normally not in any way pre-experimentally associated with a direction or location. The crucial manipulation was the visibility of the cues, which were presented either visibly or backward masked. Furthermore, to investigate the temporal dynamics of the cues’ possible effects on attention, the cue target stimulus-onset-asynchrony (SOA) varied from 100 to 600 ms. As it has been shown that the magnitude of validity effects can depend strongly on the cue target SOA (e.g., Shulman et al., 1979; Posner, 1980), and masked priming effects are likewise susceptible to the interval between masked stimuli and target (e.g., Vorberg et al., 2003), this relatively broad range of SOAs was applied, especially regarding the novelty of this research. Finally, we analyzed sequential interactions of visible and masked cues, specifically whether masked cues are able to impact on attention in the context of a previously presented visible or masked cue.

#### Participants

Twenty-six students (five males) of the University of Würzburg with an average age of 22 years participated in the experiment in fulfillment of course requirements or payment (18 Euro). All reported having normal or corrected-to-normal vision, and were not familiar with the purpose of the experiment. The experiment was completed in three sessions that were run on separate days. Each session lasted approximately 1 h.

#### Apparatus and stimuli

The experiment took place in a dimly lit room. An IBM compatible computer with a 17″ VGA-Display and the software package E-Prime^™^ (Schneider et al., 2002) were used for stimulus presentation and response sampling. Stimulus presentation was synchronized with the vertical retraces of a 100-Hz monitor. Responses were executed with the index fingers of both hands and collected with external response keys. All stimuli were presented in white on a black background. The letter *V* or *H* functioned as central cues, presented in Arial font with a size of 30 pixels. Diamonds and squares with an edge length of 2 cm served as targets and distractors, respectively. In each target display, either one target and one distractor, or two distractors were presented on the left and on the right side, with a distance of 5 cm to the center. In trials with masked cues, the forward mask and backward mask consisted of a random string of four symbols (chosen from: #, &, $, and %), presented in Arial font with a size of 40 pixels.

#### Procedure and design

**Figure 1:**
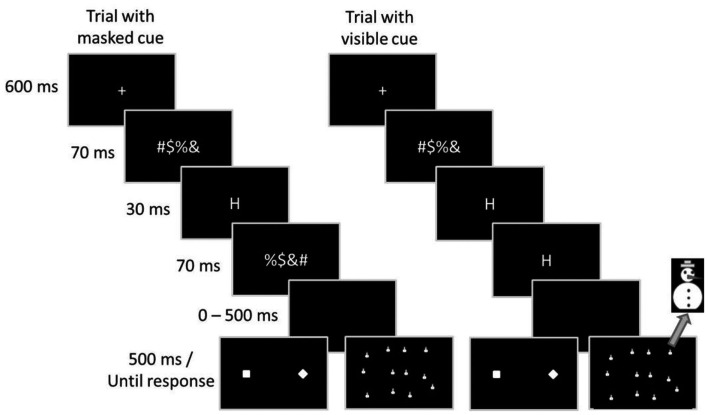
**Sequence of stimuli in Experiments 1 and 2**. On the left side, a trial with short cue duration and a backward mask is depicted. On the right side, a trial with longer cue duration and no backward mask is depicted. The left target display shows the target display of Experiment 1, with target present. The right target display depicts the search display of Experiment 2.

The sequence of events in a trial is depicted in Figure 1. Each trial started with a central fixation cross extending 0.7 cm × 0.7 cm that was presented for 600 ms. Following the fixation cross, a forward mask was presented for 70 ms. In trials with masked cues, the cue was presented for 30 ms, followed by a backward mask that was presented for 70 ms. In trials with visible cues, the cue was presented for 100 ms, and the backward mask was omitted. The target display appeared either immediately or after an interval of 100, 200, 300, 400, 500, or 600 ms and remained for 500 ms. Participants could respond within a time window of 2000 ms after target onset. After response execution a fixed time interval of 1000 ms elapsed before the next trial started.

Participants had to perform a single choice RT task. They were instructed to respond as fast as possible by pressing the spacebar when a target was present on either the left location or the right location, and not to respond when no target was present. Errors were indicated by the German word for wrong (“Falsch!”) presented in red in the lower part of the monitor. RT were recorded from the onset of the target stimulus until a response was given.

Each block of 144 trials featured 24 catch trials in which no target was present and the participants were instructed not to respond. When a target was present, the cue indicated the location of the target correctly in 96 of these trials, i.e., with a validity of 80%. Considering all trials including catch trials, this results in an overall cue validity of 67%. During each block, each possible combination of the factors visibility of the cue, identity of the cue, location of the target, and cue target-interval was presented once in the case of an invalid trial (24 trials), and four times in the case of a valid trial (96 trials), with the sequence of trials being randomly determined. The experiment consisted of three sessions (two sessions for the main experiment, one session for assessment of cue visibility) that took approximately 1 h each. Participants performed one practice block and five experimental blocks in the first two sessions.

Participants were informed that a visible cue, the letter *V* or *H*, will be presented in 50% of the trials, and that the cue predicted the correct location of the target in most trials. They were told not to move their eyes away from fixation when they shifted their attention. Eye movements were, however, not measured during the experiment, so that we cannot exclude the possibility of eye movements. Participants were not informed about the masked cues. The mapping of each cue to the left or to the right location was counterbalanced across participants.

#### Assessment of cue visibility

A visibility test consisting of 10 blocks of 72 trials each featuring both non-masked cues and masked cues constituted the third session of the experiment. Participants were fully informed about the structure of a trial and the presence of masked cues. They had to perform a forced choice discrimination task. For this task, the sequence of stimuli was exactly the same as in the main experiment. However, there was no time limit after target onset, and the overall cue validity was lowered to 50%, so that the participants could not infer from the location of the target which cue was more likely. Participants were asked to discriminate whether a *V* or an *H* was presented, and had to press one of two response keys accordingly. Participants were instructed to take their time, to try to be as accurate as possible, and if they had not seen anything to guess, bearing in mind the probability for either cue was equal.

## Results

### Experiment 1

**Figure 2:**
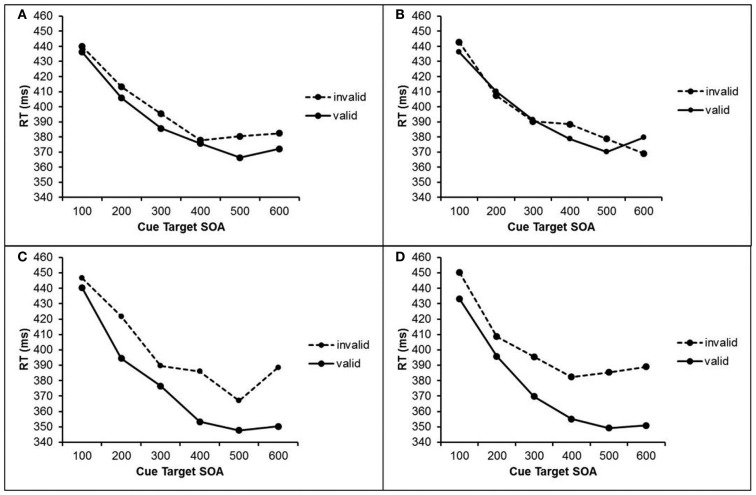
**RTs in Experiment 1 after masked cues (upper half) and visible cues (lower half) as a function of cue validity, cue target SOA, and visibility of the previous cue**. **(A)** RTs after masked cue when the previous cue was masked. **(B)** RTs after masked cues when the previous cue was visible. **(C)** RTs after visible cues when the previous cue was masked. **(D)** RTs after visible cues when the previous cue was visible.

Trials with RTs deviating more than 2.5 standard deviations (SDs) from the mean RT of each participant and each condition were excluded (1.3% of all trials). Mean RTs for correct responses were submitted to a repeated measures analysis of variance (ANOVA) with the within-subject factors cue visibility (visible cue vs. masked cue), validity (valid cue vs. invalid cue), cue target SOA (100, 200, 300, 400, 500, and 600 ms), and previous cue visibility (visible cue vs. masked cue in trial n-1). The results are depicted in Figure 2.

We found significant main effects for the factors cue validity, *F*(1, 25) = 12.5, *p* = 0.002), cue visibility, *F*(1, 25) = 6.05, *p* = 0.021, and cue target SOA, *F*(1, 25) = 122.7, *p* < 0.001. These main effects indicate faster responses after valid cues than after invalid cues (384 vs. 399 ms), faster responses after visible cues than after masked cues (388 vs. 395 ms), and faster responses with longer SOAs. The interaction of cue validity and cue visibility was significant, *F*(1, 25) = 5.51, *p* = 0.027, as well as the interaction of cue validity and cue target SOA, *F*(1, 25) = 3.58, *p* = 0.005. Additionally, the three-way interaction of cue validity, cue visibility, and previous cue visibility reached significance, *F*(1, 25) = 5.11, *p* = 0.033. To further analyze these interactions, we conducted two separate ANOVAs for trials with visible cues and trials with masked cues.

With visible cues, we found a 25 ms effect of cue validity, *F*(1, 25) = 9.28, *p* = 0.005, as well as an interaction of cue validity and cue target SOA, *F*(5, 125) = 5.05, *p *< 0.001, which reflects larger validity effects with longer SOAs. There was no interaction of cue validity and previous visibility, *F* < 1.

With masked cues, there was also a significant effect of cue validity, *F*(1, 25) = 4.61, *p* = 0.042. Participants responded 5 ms faster after valid cues than after invalid cues. Additionally, we found a marginally significant interaction of cue validity and previous cue visibility, *F*(1, 25) = 3.13, *p* = 0.089. Single comparisons revealed that validity effects of masked cues were present only after trials with masked cues, *t*(25) = 2.43, *p* = 0.023. Here, participants responded 8 ms faster after valid cue than after invalid cues. After trials with visible cues, however, no such validity effect was found *t*(25) = 0.71, *p* = 0.472.

To shed light on possible underlying mechanisms of this observation, we analyzed RTs regarding cue validity (valid vs. invalid) and cued location repetition (repetition or change compared to previous trial) separately for visible and masked cues. For masked cues that follow a visible cue, we found an interaction of cue validity and previously cued location *F*(1, 25) = 10.12, *p* = 0.004. When the cued location was the same as in the previous trial, a regular validity effect of 9 ms was present. When the cued locations changed, however, the validity effect was reversed, with RTs of 392 ms after invalid cues and RTs of 397 ms after valid cues. In other words, in both cases, RTs were shorter at the previously cued location compared to the previously non-cued location. In masked trials after masked cues, a similar interaction was present, with a larger cuing effect when the cued location remained the same (9 ms) than when it changed (3 ms), but this interaction was not significant, *F*(1, 25) = 1.47, *p* = 0.24.

For visible cues that follow a visible cue, this interaction was also significant, *F*(1, 25) = 8.61, *p* = 0.007, with a larger validity effect (33 ms) when the cued location remained the same than when it changed (18 ms). For visible cues that follow a masked cue the cuing effect was also a larger when the cued location remained the same (26 ms) than when it changed (20 ms), but this interaction missed significance, *F*(1, 25) = 1.22, *p* = 0.279.

Cue visibility was assessed by computing the signal detection measure *d*′, treating the cue *V* as signal, and the cue *H* as noise. Participants’ discrimination performance for the masked cues was *d*′ = 0.54, with a mean hit rate of 55.7% and a mean false alarm rate of 37.8%. This value deviated from zero *t*(24) = 4.64, *p* < 0.001. To test whether any validity effects with masked cues can be ascribed to cue visibility, we assessed the relationship between each participants’ individual *d*′ score and the effect of valid and invalid masked cues on RT. We adopted a procedure suggested by Greenwald et al. (1995), see also Greenwald et al. (1996), Draine and Greenwald (1998) and regressed the validity effect of each participant onto individual *d*′ scores. This analysis showed that *d*′ scores and the effects of masked cues are not significantly correlated (*r* = 0.283, *p* = 0.16), which implies that while it cannot be definitely ruled out that some masked cues were consciously perceived, the observed effects are mostly independent of individual cue visibility and are by and large not due to conscious perception of some of the cues.

## Discussion

### Experiment 1

In Experiment 1, we investigated whether centrally presented cues lead to spatial anticipations and accordant shifts of attention, and how this effect depends on the visibility of the cues, the cue target SOA, and the visibility of the previous cue. The results show that participants did form spatial anticipations based on the cues’ information and shifted their attention accordingly. With visible cues, the cueing effect increased with SOA. As the interpretation of the cue and voluntarily shifting of attention takes effort and time, the benefits of correct anticipations are more pronounced when they happen before target onset.

Remarkably, masked cues also lead to the formation of spatial anticipations and accordant shifts of attention. These anticipations based on masked cues were, however, found to be more susceptible to external modulation. Specifically, masked cues were only able to impact on attention when there were no current spatial anticipations that were induced by visible information in the previous trial, i.e., masked cues were effective only when the previously presented cue was also masked, but not when it was visible.

Previously formed spatial anticipations generally had an impact on orienting of attention. Responding was faster when the target appeared at the previously cued location than when it did not. As noted above this impact of the previously cued location was stronger when the cue in the preceding trial was visible than when it mas masked. In fact, when the previous cue was visible and the current cue was masked, the impact of the previous cuing seemed strong enough to override the cuing effect by a masked cue. We found that the validity effect was actually reversed when the previous cue indicated another location than the current masked cue. In this case, responses were faster with invalid cues, as this location was the one that was previously cued, and slower with valid cues, as the target then appeared at the previously non-cued location. In other words, orienting of attention was influenced stronger by the spatial anticipation formed in the previous trial than by the current masked cue. It is not entirely clear, however, whether the persisting spatial anticipation is in fact solely due to the previous cue’s information, or whether it is also influenced by the previous target location. Possibly, participants tended to orient their attention toward the previous target location. Effects of the previous target location might be disentangled from effects of the previous cued location by additionally analyzing the cue validity in the previous trial. Unfortunately, the experimental design at hand does not allow for a statistically sound analysis with this additional factor, as particular factor combinations yield too few cases for each participants to perform a meaningful analysis. Thus, the distinct role of the previous target location in the sequential modulation cannot be clarified with the data at hand.

The fragility of the masked cueing effect could explain previously unsuccessful efforts to find this effect. In McCormick’s (1997) study, the exogenous shift of attention triggered by the peripherally presented cue might have suppressed an effect of spatial anticipation (which would be directed on the opposite side of the screen), as an anticipation that is based on a masked cue can be influenced and possibly suppressed by other spatial information currently present.

## Material and Methods

### Experiment 2

Experiment 1 showed that anticipative shifts of attention can be triggered by masked symbolic cues. To replicate and further elaborate this finding, a more demanding visual search task with a two forced choice RT task was implemented in Experiment 2 instead of the elementary target detection task of Experiment 1. To this end, Experiment 2 featured a visual search display with 11 distractors and 1 target. Here, participants had to search for one of two possible targets amongst several stimuli. The difficulty to find the target and to identify it was therefore far higher than in Experiment 1, and the benefits and costs after valid and invalid cues, respectively, were thus supposedly much larger. Like in Experiment 1, we varied the visibility of the cue and the cue target SOA. Also, we again analyze sequential effects of cue visibility.

#### Participants

Twenty-one students (13 males) of the University of Würzburg with an average age of 24 years participated in the experiment in fulfillment of course requirements or for payment (18 Euro). Informed consent was obtained from all participants. All participants reported having normal or corrected-to-normal vision, and were not familiar with the purpose of the experiment. The experiment was completed in three sessions that were run on separate days. Each session lasted approximately 1 h.

#### Apparatus and stimuli

The experiment took place in a dimly lit room. An IBM compatible computer with a 17″ VGA-Display and the software package E-Prime^™^ were used for stimulus presentation and response sampling. Stimulus presentation was synchronized with the vertical retraces of a 100-Hz monitor. Responses were executed with the index fingers of both hands and collected with external response keys. All stimuli were presented on a black background. Cue stimuli and masking stimuli were identical to those in Experiment 1. The target display consisted of 12 snowmen (extending 8 mm × 18 mm) wearing colored hats (extending 7 mm × 3 mm; see lower right screen of Figure 1). The snowmen were quasi-randomly distributed over the screen. For this, the screen was subdivided in 4 × 3 grids (invisible to the participants), and in each grid a snowman was presented at a random location, so that six snowmen were presented on locations on the left half of the screen, and six snowmen were presented on locations on the right side of the screen. There was always exactly one target snowman present, which was denoted by wearing either a blue hat or a gray hat. The other 11 distractor snowmen wore red, violet, orange, yellow, and green hats.

#### Procedure and design

The sequence of events in a trial until the target display was identical to that of Experiment 1 (see Figure 1). The target search display was presented until a response was given, with no limitation by a response window. After response execution a fixed time interval of 1000 ms elapsed before the next trial started.

Participants had to perform a two forced choice RT task. They were instructed to respond as fast as possible to the color of the target snowman’s hat. The mapping of left and right responses to gray vs. blue hats was counterbalanced across participants. Errors were indicated by the German word for wrong (“Falsch!”) presented in red in the lower part of the monitor. RT were recorded from the onset of the target stimulus until a response was given.

Participants were informed that a visible cue is presented in 50% of the trials, and that the cue indicates the correct side of the screen (left vs. right) where the target appears in most trials. They were not informed about the masked cues. The mapping of each cue to the left or to the right side of the screen was counterbalanced across participants.

Each of two experimental sessions consisted of one short training block (20 trials) and nine experimental blocks of 80 trials each. All 96 possible combinations of cues, visibility of the cue, target location (left or right), cue target SOA, and target identity (either blue or gray hat) were presented within the span of three blocks. All combinations with invalid cues were presented once within three blocks, and all combinations with valid cues were presented four times within three blocks. The overall cue validity was thus 80%. The experiment consisted of three sessions that took approximately 1 h each.

#### Assessment of cue visibility

A visibility test consisting of six blocks of 96 trials featuring both non-masked cues and masked cues was applied in the third experimental session. Participants were fully informed about the structure of a trial and the presence of masked cues. They had to perform a forced choice discrimination task. For this task, the sequence of stimuli was exactly the same as in the main experiment. However, the overall cue validity was lowered to 50%, so that the participants could not infer from the location of the target which cue was more likely. Participants were asked to discriminate whether a *V* or an *H* was presented, and had to press one of two response keys accordingly. Participants were instructed to take their time, to try to be as accurate as possible, and if they had not seen anything to guess, bearing in mind the probability for either cue was equal.

## Results

### Experiment 2

**Figure 3:**
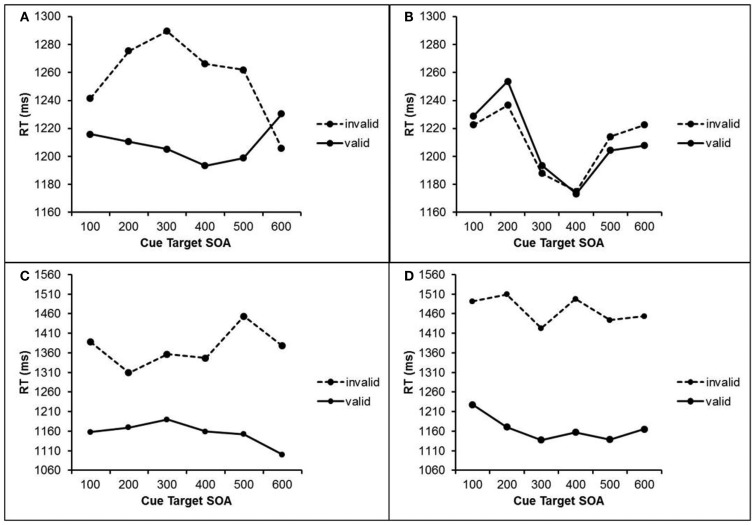
**RTs in Experiment 2 after masked cues (upper half) and visible cues (lower half) as a function of cue validity, cue target SOA, and visibility of the previous cue**. **(A)** RTs after masked cue when the previous cue was masked. **(B)** RTs after masked cues when the previous cue was visible. **(C)** RTs after visible cues when the previous cue was masked. **(D)** RTs after visible cues when the previous cue was visible.

Trials with RTs deviating more than 2.5 SDs from the mean RT of each participant and each condition were excluded (1.7% of all trials). RT data were submitted to a repeated measures ANOVA with the within-subject factors cue visibility (visible cue vs. masked cue), validity (valid cue vs. invalid cue), cue target SOA (100–600 ms), and previous cue visibility (visible cue vs. masked cue in trial n-1). The results are depicted in Figure 3.

This analysis revealed a main effect of validity, *F*(1, 20) = 24.2, *p *< 0.001. Participants responded faster after valid cues (1185 ms) than after invalid cues (1327 ms) The main effect of cue visibility was also significant, *F*(1, 23) = 15.8, *p* = 0.001. Participants responded faster (1221 ms) after masked cues than after visible cues (1291 ms). The interaction of cue visibility and validity was significant, *F*(1, 20) = 17.0, *p* = 0.001, as was the three-way interaction of cue visibility, validity, and previous cue visibility, *F*(1, 20) = 13.0, *p* = 0.002. No other main effects or interactions were significant (*p*s > 0.166). To further investigate these interactions, we conducted two separate ANOVAs for visible and masked cues.

With visible cues, a main effect of validity revealed faster responses after valid (1164 ms) than after invalid (1440 ms) cues, *F*(1, 20) = 21.2, *p *< 0.001. The interaction of validity and previous cue visibility was significant, *F*(1, 20) = 11.3, *p* = 0.003. Here, validity effects were larger after trials with visible cues (303 ms) than after trials with masked cues (217 ms).

With masked cues, we also found a significant main effect of validity, *F*(1, 20) = 4.84, *p* = 0.04, with responses that were 24 ms faster after valid than after invalid masked cues. The interaction of validity and previous visibility just failed to reached marginal significance, *F*(1, 20) = 2.84, *p* = 0.11. In contrast to visible cues, masked cues only impacted on attention when the previous cue was also masked, *t*(20) = 2.38, *p* = 0.027 which is reflected in a validity effect of 48 ms. When following a visible cue, masked cues were not able to impact on attention at all, evident by an absent validity effect (0 ms).

To further understand these sequential effects we analyzed RTs regarding cue validity (valid vs. invalid) and cued location repetition (repetition or change compared to previous trial) separately for visible and masked cues. For masked cues that follow a visible cue, we found an interaction of cue validity and repetition of cued location, *F*(1, 20) = 7.35, *p* = 0.013. Similar to Experiment 1, there was a regular cuing effect of 72 ms when the cued locations repeated, which was reversed to a negative cuing effect (−58 ms) when the cued location changed. For masked cues that follow a masked cue, the interaction was marginally significant, *F*(1, 20) = 3.27, *p* = 0.086, and also reflected a regular cuing effect when the cued location repeated (77 ms) and a reversed cueing effect when the location switched (−3 ms).

For visible cues that follow a masked cue, the interaction was also significant, *F*(1, 20) = 14.48, *p* = 0.001, with a larger cuing effect (278 ms) when the cued location repeated than when it changed (150 ms). However, no significant interaction was found for visible cues that follow a visible cue, *F *< 1.

Cue visibility was assessed by computing the signal detection measure *d*′, treating the cue *V* as signal and the cue *H* as noise. Participants’ discrimination performance for the masked cues was *d*′ = 0.186, with a mean hit rate of 54.5% and a mean false alarm rate of 47.4%. This value deviated from zero *t*(20) = 2.48, *p* = 0.023. To test whether any validity effects of masked cues can be ascribed to cue visibility, we assessed the relationship between each participants’ individual *d*′ score and the effect of valid and invalid masked cues on RT. Following a procedure suggested by Greenwald et al. (1995), see also Draine and Greenwald (1998), Greenwald et al. (1996) and regressed the validity effect of each participant (RT invalid trials – RT valid trials) onto individual *d*′ scores. This analysis showed that *d*′ scores and the effects of masked cues were not significantly correlated (*r* = 0.126, *p* = 0.596), which implies that the observed effects are mostly independent of individual cue visibility and are by and large not due to conscious perception of some of the cues.

## Discussion

### Experiment 2

The results of Experiments 2 confirmed the findings of Experiment 1 in a visual search context: participants are able to form spatial anticipations and shift their attention accordingly on the basis of both visible and masked centrally presented cues. Participants shifted their attention to the side where they anticipated the target, which resulted in shorter RTs when the target was in fact amongst the stimuli on this side of the screen, and in longer RTs when the target was actually on the other side of the screen. With visible cues, this resulted in responses that were 276 ms faster after valid than after invalid cues. With masked cues, this effect was much smaller (24 ms) but still present. This shows that even cues that we are not aware of are able to induce spatial anticipations that lead to according shifts of attention. However, an effect of masked cues was found only when the previous trial did not contain a visible cue. This observation suggests that information provided by masked stimuli takes effect only when no stronger spatial information, i.e., that of visible stimuli, is in a still active state. The cuing effect depended on the previously cued location. It was stronger when the cued locations repeated from previous to current trials than when they switched. As in Experiment 1, the impact of a previous visible cue was strong enough to invert the regular cuing effect from a current masked cue. Yet, even previous masked cues were able to modify cuing effects in the current trial to some degree. Within this regard, it again remains unclear whether the location of the previous target additionally influenced orienting of attention in the current trial. One exception from this overall pattern, which otherwise emerged quite consistently in both experiments, was the lack of sequence effects with two subsequent visible cues in Experiment 2. At present we have no obvious explanation for this.

The cue target SOA had less of an influence than in Experiment 1, probably because of the different time frame of the tasks. Conceivably, the information provided by the cue was not effectively used with very short cue target SOAs in Experiment 1 due to RTs that were shorter than the time needed to interpret the cue and shift one’s attention. When the target display appeared shortly after the cue and probably before the shift of attention was initiated, the simple task was carried out before the accordant shift of attention was performed. With the visual search task in Experiment 2, the target display could appear before the shift of attention was initiated, but the information provided by the cue could still be effectively used because of the rather long search RTs to find the target.

## General Discussion

The ability to shift our attention in anticipation of future events is an elementary process of cognitive control. Here, we provided evidence that such shifts of attention can be elicited by masked cues.

In two experiments, centrally presented letter cues informed the participants about the likely location of the upcoming target. Participants responded faster when the target appeared at the anticipated location (i.e., after a valid cue) rather than at another location (i.e., after an invalid cue). This indicated that participants formed spatial anticipations regarding the location of the target and shifted their attention accordingly. Strikingly, this was true for visible as well as for masked cues. This is especially remarkable as the cues were deliberately chosen to be spatially arbitrary. Unlike arrows, letters possess no inherent spatial meaning. Thus, letters have to be interpreted regarding their spatial meaning to form spatial anticipations. The observed effects of the cues therefore cannot be attributed to automatically induced shifts of attention that are based on over learned spatial relations like in previous studies (Reuss et al., 2011b; Al-Janabi and Finkbeiner, 2012), but must be attributed to anticipatory shifts of attention generated endogenously.

In Experiment 1, participants had to recognize whether the target display contained one distractor and one target, or two distractors. With this single choice task, RT were very short. Consequently, the cue target SOA modulated the validity effect. Only with longer SOAs, the spatial information provided by the cue could be used effectively to orient attention before the target occurred and the response was given. This interaction was more pronounced in the visible cue condition. Most importantly, however, the validity effects were found both for visible and masked cues. The latter, however, were only able to impact on attention when the previous cue was also masked. This indicates that visible cues lead to strong spatial anticipations that are able to persist at least until the next trial and interfere with forming new spatial anticipations, especially those based on masked cues.

In Experiment 2, participants had to actually search for the target among eleven distractors. Thus, target detection was harder and RT were longer than in Experiment 1. The increased task difficulty worked as an incentive to use the cues, as the information provided by the cues is potentially more beneficial the harder the target is to detect, which resulted in large effects of cue validity. Also, the influence of the cue target SOA was reduced in Experiment 2 compared to Experiment 1, so that effects of cue validity were also present with very short SOAs. Besides that, the overall pattern of results was very similar to Experiment 1. Again, validity effects were found both for masked cues and visible cues, and the effects of masked cues strongly depended on the visibility of the previous cue: when the previous cue was visible, no effects of a masked cue could be observed at all. Masked cues were effective only after trials with masked cues.

To further investigate the underlying mechanism of these sequential modulations, we analyzed whether the cue information of the previous trial interacts with the current cue information depending on their respective visibility. These analyses revealed for both experiments that when the current cue was masked, participants oriented their attention toward the location that was previously cued if this previous cue was visible (and to a lesser extent also when the previous cue was masked). When the same location as in the previous trial was cued, participants responded faster when the target appeared at the cued than at the non-cued location. However, when the currently cued location differed from the cued location in the previous trial, participants responded actually faster when the target appeared at the currently non-cued location than at the currently cued location. In other words, responses were faster when the target appeared at the location indicated by the previous visible cue compared to when the target appeared at the location that was not indicated by the previous visible cue, whereas the current masked cue had no substantial impact. This strongly suggests that spatial anticipations persist until the next trial and still influence the orienting of attention to an extent that nullifies effects of masked cues (in the case of a previous visible cue) or at least modulates their effect (in the case of a previous masked cue). It is also plausible, however, that not only the information of the previous cue, but also the actual target location in the previous trial influenced the spatial anticipation that carried over to the next trial. As the cued location is identical to the target location in the majority of trials, the observed effects can be due to either of these factors. While we analyzed whether the visibility of the previous cue influenced the observed validity effects, previous cue information is confounded with previous target location because cues were valid in most trials. Unfortunately, the present data set does not allow us to soundly disentangle the effects of both the cue information and the target location on orienting of attention in the subsequent trial, as particular factor combinations in the necessary analysis occur too infrequently to enable a meaningful analysis.

The persisting effect of already active spatial anticipations is strong enough to still impact on attention even when a visible cue is presented. However, spatial anticipations that are induced by visible cues are more resilient to such influences, so that their effect is merely modulated by already active spatial anticipations, but not completely overridden. Interestingly, this modulation took place even when the previous cue was masked.

The observation that even masked cues lead to anticipatory shifts of attention is remarkable because endogenous orienting of attention is regarded as one of the most elementary processes of cognitive control, and cognitive control processes are traditionally associated with consciousness (e.g., Atkinson and Shiffrin, 1968; Norman and Shallice, 1986), and “authors speak of “conscious control” as if there could be no alternative” (Hommel, 2007, p. 161). An effect of masked cues thus casts doubt on this proposed correlation. As outrageous as this devaluation of the functionality of our consciousness may seem, given its antagonism to our introspective impression that our conscious will controls our actions (Wegner, 2002), it is in line with recent findings concerning the relation of consciousness and other cognitive control processes like inhibitory processes (van Gaal et al., 2008, 2009; Hughes et al., 2009) and task set activation (Mattler, 2006; Lau and Passingham, 2007; Reuss et al., 2011a). These cognitive control processes were shown to be able to work outside of awareness.

However, one should not dismiss the differences that exist between the effects of visible cues and masked cues. First, there are quantitative differences when looking at the benefits and costs of valid cues and invalid cues depending on their visibility. The effects of visible cues are distinctively larger than those of masked cues. This indicates a stronger and more reliable impact on cognitive control processes than the one provided by masked cues. Such a quantitative difference was, for example, also found by Reuss et al. (2011a) regarding the activation of task sets by masked cues. Second, the effects of visible cues are less prone to potential interference than the effects of masked cues. In the two experiments presented here, this is illustrated by the impact of cues on attention in the next trial. In both experiments, spatial anticipations induced by visible cues were still active in the subsequent trial. In trials with masked cues, this persisting spatial anticipation was able to strongly influence the orienting of attention, sometimes to an extent that the current cue had no noticeable effect on attention. Visible cues were in contrast more robust against such a persisting influence. Persisting spatial anticipations were able to impact on attention in trials with visible cues as well, but the effect of visible cues was strong enough to also significantly impact on attention.

To conclude, we showed in two experiments that spatial anticipations and corresponding shifts of attention are able to be induced both by visible cues and by masked cues. This observation challenges the notion of a strong link between orienting of attention as a prototypical control process and consciousness. However, awareness of the cue still played a role regarding the reliability and robustness of the control process.

## Conflict of Interest Statement

The authors declare that the research was conducted in the absence of any commercial or financial relationships that could be construed as a potential conflict of interest.
